# Ultrasonic Assisted Extraction of Quinoa (*Chenopodium quinoa* Willd.) Protein and Effect of Heat Treatment on Its In Vitro Digestion Characteristics

**DOI:** 10.3390/foods11050771

**Published:** 2022-03-07

**Authors:** Xingfen He, Bin Wang, Baotang Zhao, Fumin Yang

**Affiliations:** College of Food Science and Engineering, Gansu Agricultural University, Lanzhou 730070, China; hexingfen163@163.com (X.H.); wangbin_1519@163.com (B.W.); zhaobaotang@126.com (B.Z.)

**Keywords:** quinoa protein, ultrasounic-assisted extraction, in vitro digestion, amino acid content, nuclear magnetic resonance hydrogen spectroscopy

## Abstract

To extract and utilise the protein in quinoa efficiently, we investigated the effect of rate of quinoa protein isolate (QPI) extraction by ultrasound-assisted alkaline extraction and traditional alkaline extraction methods using single-factor experiments and Box-Behnken design. The effect of different heat treatment temperature and time on QPI functional properties and in vitro digestion characteristics were also investigated. The results showed that the optimal conditions of ultrasound- assisted alkaline extraction process were: ultrasonic time 99 min, solid-liquid ratio 1:20 *w:v*, ultrasonic temperature 47 °C, and pH 10, and its extraction rate and purity were 74.67 ± 1.08% and 87.17 ± 0.58%, respectively. It was 10.18% and 5.49% higher than that of the alkali-soluble acid precipitation method, respectively. The isoelectric point (pI) of QPI obtained by this method was 4.5. The flexibility and turbidity of QPI had maximum values at 90 °C, 30 min, and 121 °C, 30 min, which were 0.42 and 0.94, respectively. In addition, heat treatment changed the 1.77–2.79 ppm protein characteristic region in QPI’s nuclear magnetic resonance hydrogen spectroscopy (^1^H NMR). After heating at 90 °C and 121 °C for 30 min, the hydrolysis degree and total amino acid content at the end of digestion (121 °C, 30 min) were significantly lower than those of untreated QPI by 20.64% and 27.85%. Our study provides basic data for the efficient extraction and utilisation of QPI.

## 1. Introduction

Quinoa (*Chenopodium quinoa* Willd.), also known as quinoa flour or grey rice. Its seeds are rich in high-quality protein and have high nutritional value [1]. In particular, the growing popularity of quinoa seeds is due to the rich content of proteins, dietary fibers, B vitamins, and dietary minerals as well as for being a gluten-free food [2]. It is a perfect “whole food” for humans and is listed as one of the top ten healthy nutritional foods globally [3]. Quinoa protein isolate (QPI) is widely used in the food industry for preparation of infant foods, edible films, beverages, sauces, and sausages due to its gluten-free nature [4]. In addition, quinoin (a type 1 ribosome-inactivating protein) is contained in quinoa seeds. Quinoin is considered a toxic protein present in both quinoa seeds and sprouts, which is resistant to both heat treatment and in vitro digestion [5]. Therefore, it is necessary to treat quinoa seeds by heating before consumption.

The traditional method of quinoa protein extraction mainly involves alkaline solubilisation and acid precipitation. However, due to disadvantages such as low extraction rate, long extraction time, and ability to change the structure and properties of the active material, the application of this traditional method for QPI extraction is limited. Ruiz et al. [6] demonstrated that the thermal stability of QPI worsened with an increase in pH at the time extraction. Guerreo-Ochoa et al. [7] reported that the yield of QPI obtained by the traditional alkaline extraction method was only 62.1%. Literature suggests that ultrasound, due to its cavitation properties, can shorten the extraction time and increase the extraction rate of proteins [8]. Additionally, improvement in solubility was reported for rice protein extracted by ultrasound-assisted alkaline extraction method [9]. The optimal ultrasound-assisted extraction conditions for rice bran protein were: amplitude 76%, extraction time 18 min, solid-liquid ratio 0.99 g/10 mL; and the protein yield was 4.73 ± 0.03% [10]. Moreover, in comparison with the traditional extraction method, ultrasound-assisted method increased the protein yield and protein content of duck liver by 67.7% and 4.6%, respectively [8].

The structural and functional properties of proteins play an important role in food processing and determine the application range of proteins. Therefore, research on the effects of different treatments on the structural and functional properties of food proteins is essential to better understand their roles in food systems. Various treatments such as heat [11], ultrasound [12], high pressure [13], and pH [14] significantly affect the structural and functional properties of proteins, among which the heat treatment has been one of the major methods to modify the characteristics of food ingredients and has a great impact on the structural and functional properties of proteins [15]. What’s more, most food proteins undergo structural transformation during heat processing which could affect the digestion of proteins [16]. The heat treatment reduced the intermolecular forces of soluble proteins and increased flexibility [17]. Protein from Pacific oyster showed higher digestibility at relatively low temperature, but lower digestibility at relatively high temperature [18].

Although previous studies have documented the effects of heat treatment on the in vitro digestion characteristics of pea protein [19], whey protein [20], and soybean protein [21], there are few references about studies on the optimisation of ultrasounic-assisted alkaline extraction of QPI and the effect of heat treatment on the in vitro digestion characteristics of QPI. Therefore, this study aimed to (1) determine the optimum process conditions for ultrasound-assisted alkaline extraction of quinoa protein; (2) compare the optimum process conditions, extraction rate, and purity of QPI obtained by ultrasound-assisted alkaline extraction process and alkali-solution and acid-isolation extraction process; (3) investigate the effect of different heat treatment time and temperature on structural and functional properties of QPI; and (4) determine the effect of heat treatment on its in vitro digestion characteristics.

## 2. Materials and Methods

### 2.1. Materials

Quinoa was planted in the open field at Wanggeertang town (East longitude 102.847758, North latitude 35.216012, Altitude 2500 m), Xia he County, Gan nan Tibetan Autonomous Prefecture, China in May. Commercial mature quinoa was harvested in September of the same year. Within 3 h of mechanical shelling, quinoa was packed in woven bags and transported to the laboratory. Samples were stored at 4 °C for later use. Pepsin from porcine gastric mucosa (power, ≥250 units/mg solid) was purchased from Sigma-Aldrich (St. Louis, MO, USA). Pepsin (Potency: 1:3000) was purchased from Shanghai Yuanye Biological Technology Co., Ltd., (Shanghai, China). The rest of the chemicals used are of analytical grade.

### 2.2. Extraction of Quinoa Proteins

Extraction of quinoa proteins was carried out as described by Silventoinen, & Sozer [22]. The quinoa seeds was crushed, sifted using a 60-mesh sieve, and degreased with petroleum ether (Auto fat analyzer, Sex 406, Jinan Haineng Instrument Co., Ltd., Jinan, China). The degreased quinoa flour was air-dried followed by addition of distilled water to obtain a certain solid-liquid ratio. The pH was adjusted using 1 M NaOH followed by extraction of quinoa proteins at a set ultrasonic temperature. The extract was then centrifuged for 15 min at 6369× *g* (H−1850R, Changsha Xiangyi Centrifuge Co., Ltd., Changsha, China). And the supernatant was collected. The pH of the supernatant was adjusted to isoelectric point of QPI using 1 M HCl and incubated at 4 °C for 120 min. After that, the extract was centrifuged for 15 min at 6369× *g* to collect the precipitate. The precipitate was resuspended in distilled water and washed 3–5 times. The pH was adjusted to 7.0 using 1 M NaOH. QPI was obtained by vacuum freeze drying the resuspended precipitate in a vacuum freeze-drying machine (LyoQuest-85, Telstar Lab, Barcelona, Spain) and stored at −20 °C until use.

### 2.3. Determination of Extraction Rate and Purity of QPI

The extraction rate and purity of QPI were determined according to the method described by Ruiz et al. [1]. The protein content refered to the method of Miller et al. [23]

(1) $$\mathrm{The}\;\mathrm{extraction}\;\mathrm{rate}\;\mathrm{of}\;\mathrm{QPI}\;\left(\%\right)=\frac{\mathrm{Isolate}\;\mathrm{protein}\;\mathrm{content}\;\left(\%\right)\times \;\mathrm{isolate}\;\mathrm{weight}\;\left(g\right)}{\mathrm{flour}\;\mathrm{protein}\;\mathrm{content}\;\left(\%\right)\times \mathrm{flour}\;\mathrm{weight}\;\left(g\right)\;}\times 100$$

(2) $$\mathrm{The}\;\mathrm{purity}\;\mathrm{of}\;\mathrm{QPI}\;\left(\%\right)=\frac{\mathrm{Isolate}\;\mathrm{protein}\;\mathrm{content}\;\left(\%\right)\times \;\mathrm{isolate}\;\mathrm{weight}\;\left(g\right)}{\mathrm{isolate}\;\mathrm{weight}\;\left(g\right)\;}\times 100$$

### 2.4. Optimisation of Quinoa Protein Extraction Process

#### 2.4.1. Single-Factor Test for Ultrasound-Assisted Alkaline Extraction

The basic conditions for extraction were: ultrasonic time 90 min, solid-liquid ratio 1:15, ultrasonic temperature 45 °C, and pH 10; for single-factor test, one of these parameters was changed at a time keeping the other three parameters constant. The different conditions tested were ultrasonic time (30, 60, 90, 120, 150 and 180 min), solid-liquid ratio (1:5, 1:10, 1:15, 1:20, 1:25 and 1:30; *w:v*), ultrasonic temperature (25, 35, 45, 55, 65 and 75 °C) and pH (8.0, 9.0, 10.0, 11.0, 12.0 and 13.0), with extraction rate of QPI as the index. The experiments were performed in triplicates.

#### 2.4.2. Response Surface Optimisation Test

On the basis of the single-factor test, the four parameters for quinoa protein extraction including ultrasonic time (A), solid-liquid ratio (B), ultrasonic temperature (C) and pH(D) were optimised. The response surface test scheme was designed according to the Box-Behnken method in the Design-Expert 8.0.6 software, as shown in Tables S1 and S2.

#### 2.4.3. Comparison between Ultrasound-Assisted and Traditional Alkaline Extraction Methods

The extraction rate and purity of QPI obtained by the ultrasound-assisted alkaline extraction method and the traditional alkaline extraction method were compared and the optimum technological conditions were determined.

### 2.5. Isoelectric Point

The isoelectric point of QPI was determined according to the method of Pedroche et al. [24]. Under the optimal extraction conditions of QPI, eight equal portions of protein extract supernatant were collected, and the pH was adjusted to 3.0, 3.5, 4.0, 4.5, 5.0, 5.5, 6.0, 6.5 with 1 M HCl, respectively. The optimum pH of acid precipitation was determined by measuring the extraction rate of the protein. The pH corresponding to the maximum protein extraction rate is the isoelectric point of QPI.

### 2.6. Heat Treatment

QPI was dissolved in phosphate buffer (0.2 M, pH 7.0), while stirring for 2 h at ambient temperature (25 °C) and stored it overnight at 4 °C to enhance hydration. The QPI solution with a concentration of 5% was treated at different temperatures of 60, 70, 80, 90, 100, and 121 °C for 5, 10, 20, and 30 min (Employed each temperature at different times), and then quickly cooled with ice water for 5 min. Samples were stored at 4 °C and generally analyzed within 2 d of treatment [25].

### 2.7. Flexibility and Turbidity Measurement

Trypsin solution (250 μL, 0.1%) was mixed with heat-treated QPI solution (14 mL, 0.1%), and after 5 min incubation at 38 °C, 4 mL of trichloroacetic acid solution (5%) was added. After centrifugation, the supernatant was taken, and its absorbance was measured at 280 nm, indicating flexibility [26]. The heat-treated QPI solution was diluted to 3 mg/mL, and the turbidity was measured by measuring its absorbance at 400 nm after shaking and mixing [27].

### 2.8. Samples Preparation for Proton Nuclear Magnetic Resonance (^1^H NMR) Spectra Acquisition

Freeze-dried QPI (20.0 mg) was placed in a 2 mL sample tube with 1000 μL CD_4_O aqueous solution (CD_4_O:D_2_O = 1:1, *v:v*; TMSP 0.5 mM). The mixed solution was placed in a tissue grinder for 300 s (70 Hz), repeated 2 times and then centrifuged (10 min, 12,738× *g*) 2 times. The supernatant (540 μL) was transferred into a nuclear magnetic tube for 1H NMR test on a 600 MHz nuclear magnetic resonance spectrometer (AVANCE III Brook (Beijing) Technology Co., Ltd., Beijing, China). The test conditions were: constant temperature of 298 K (25 °C), pulse interval D1 of 4.00 s, spectral width of 12,335.526 Hz, and 64 samples, using a ZGPR pulse sequence to suppress the water peak. The raw spectra were imported into MestReNova software for segment integration and normalization at 0.51 ppm [28].

### 2.9. In Vitro Simulation of Gastrointestinal Digestion of QPI

#### 2.9.1. Preparation of Simulated Gastrointestinal Fluid

Simulated gastricfluid (SGF) and simulated intestinalfluid (SIF) were prepared following the harmonized protocol [29]. SGF: pepsin (0.3 g), NaCl (2.0 g) and HCl (12 M, 0.7 mL) were mixed and made up to 100 mL, and the pH of the solution was adjusted to 1.2. SIF: After KH_2_PO_4_ (0.68 g) was completely dissolved in deionized water (25 mL), NaOH solution (0.2 m, 19 mL), deionized water (40 mL) and trypsin (4.0 g) were successively added, and the volume was fixed to 100 mL. The pH of the solution is 7.5.

#### 2.9.2. In Vitro Gastrointestinal Digestion

After the heat-treated QPI solution (Treatment at 90 °C and 121 °C for 30 min respectively), SGF and SIF were incubated at 37 °C for 10 min, equal volumes of QPI solution and SGF were taken into a centrifuge tube. The centrifuge tube was placed in a water bath shaker at 37 °C, and the digested products with reaction times of 0, 30, 60, 90 and 120 min were respectively taken into the test tube, and immediately boiled at 100 °C to kill the enzyme and cooled for later use. The pH of the remaining solution in the centrifuge tube was adjusted to 7.5, an equal volume of SIF was added, and the centrifuge tube was placed in a water bath shaker at 37 °C, and the digested products with reaction times of 150, 180, 210, 240 and 270 min were respectively taken into the test tube, and immediately boiled at 100 °C to kill the enzyme and cooled for later use [30].

#### 2.9.3. Degree of Hydrolysis

The hydrolysis degree of QPI was determined by o-phthalaldehyde (OPA) method. The hydrolyzed supernatant (400 μL) was mixed with OPA (3 mL), and the absorbance was measured at 340 nm immediately after 2 min of reaction. Deionized water and serine standard solution (0.9516 mM) were used as blank and stand, respectively [31]. The degree of hydrolysis was calculated as follows:

(3) $$SerineNH_{2}=\frac{OD_{sample}-OD_{blank}}{OD_{stand}-OD_{blank}}\times 0.9516\times \frac{N\times V}{X\times P}\;$$

where *SerineNH_2_* is the serine content per gram of protein, mmol/g, *X* is the quality of the sample, g, *p* is the protein content of the sample, %, *N* is dilution times, *V* is volume of supernatant, L.

(4) $$DH\;\left(\%\right)=\frac{\left(SerineNH_{2}-\beta \right)/\alpha }{h_{tot}}\times 100$$

where *β* and α are constants 0.4 and 1, respectively, *h_tot_* is number of peptide bonds of protein, mmol/g, the *h_tot_* of quinoa protein is 7.4 mmol/g.

#### 2.9.4. Total Amino Acid Content of In Vitro Digestion Products

The enzymolysis solution (2 mL) was mixed with 6 M HCl (8 mL) and placed in a digestive tube then filled with high-purity nitrogen for 3 min. The bottle plug was quickly sealed and digested at 110 °C for 24 h. It was taken out after cooling. All the hydrolysates were transferred to 25 mL volumetric flask. The digestive solution (2 mL) was dried in an oven at 60 °C, and a few solid or stains were left at the bottom. The residue was redissolved with 2 mL of ultrapure water, then dried again, repeated for 3 times, and diluted to 5 mL. Digestion solution (2 mL) was filtered with 0.22 μm aqueous membrane into sample bottles for automatic amino acid analyzer (LA8080 Hitachi High-Tech Science Co., Ltd. Naka Office, Tokyo, Japan) testing [32].

### 2.10. Data Analysis

Experimental data are presented as mean ± standard deviation (calculated by Excel 2007). Origin 2021 was used for plotting graphs. SPSS 17.0 was used for one-way ANOVA and multiple comparisons. The response surface results were analysed and graphs were plotted using Design-Expert 8.0. MestReNova was used to analyze the ^1^H NMR spectrum.

## 3. Results and Discussion

### 3.1. Single-Factor Test Results

**Figure 1:**
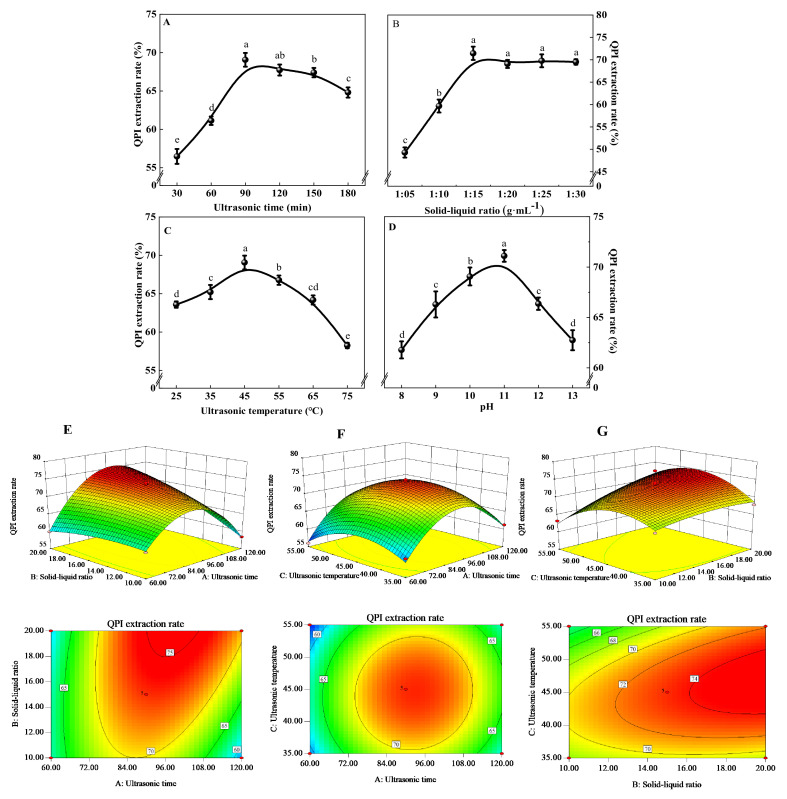
The effects of ultrasonic time (**A**), solid-liquid ratio (**B**), ultrasonic temperature (**C**), pH (**D**) and the response surface methodology and contour plots for the effects of various factors (**E**–**G**) on the extraction rate of QPI. Different letters (a–e) represent significant differences (*p* < 0.05).

Quinoa protein extraction rate increased sharply with prolongation of ultrasonic time and reached a maximum value of 69.07 ± 0.89% at 90 min, and then decreased gradually (Figure 1A). When the ultrasonic time was between 30 and 90 min, cavitation and thermal effects of the ultrasonic wave caused the solvent molecules to rapidly enter the solid matter, resulting in quick dissolution of QPI and increase in extraction rate. When the ultrasonic time exceeded 90 min, the protein structure was destroyed and degraded due to long-term cavitation and thermal effects of the ultrasound. This resulted in protein folding and aggregation due to excessive increase in hydrophobicity, leading to a decline in the dissolution rate of QPI [33]. Therefore, the optimum ultrasonic time was 60–120 min.

With an increase in the amount of solvent, quinoa protein extraction rate rapidly increased initially, reached a maximum value of 71.44 ± 1.49% when the solid-liquid ratio was 1:15, and then decreased slowly (Figure 1B). This trend is observed because the quinoa particles do not fully collide with each other under the action of ultrasonic cavitation when the concentration of quinoa is low. Appropriate solid-liquid ratio enables the quinoa particles to fully accept the force of emptiness and collide strongly with each other, which increases the quinoa protein dissolution and extraction rate [34]. However, excessive quinoa concentration will affect the mass and heat transfer effect of the material, thereby affecting the cavitation effect and decreasing the quinoa protein extraction rate. Therefore, the optimum solid-liquid ratio was 1:10–1:20.

As the ultrasonic temperature increased, quinoa protein extraction rate gradually increased, reached a maximum value of 69.07 ± 0.89% at 45 °C, and then decreased rapidly (Figure 1C). This occurs because within a certain range, the increase in temperature is conducive to the acceleration of thermal motion inside the molecule, which increases the protein dissolution and extraction rate. However, very high temperatures damage the integrity of the protein structure and reduce the dissolution and extraction rate [35]. Therefore, 35–55 °C was considered as the optimum temperature range.

With increase in pH, quinoa protein extraction rate gradually increased, reached a maximum value of 71.10 ± 0.58% at pH 11.0, and then decreased rapidly (Figure 1D). It was observed that pH changes significantly affected the extraction rate of quinoa protein (*p* < 0.05). This is mostly likely due to the loosening of tight cell structure and destruction of the protein structure in an alkaline environment, thereby increasing the amount of protein eluted [36]. As the pH increased above 11.0, the extraction rate of quinoa protein decreased, which might be due to the destruction of secondary bonds in the protein by strong bases, resulting in denaturation of the protein secondary and tertiary structure [37]. However, previous studies have shown that the higher the pH when extracting QPI, the worse the thermal stability, and the purity of QPI is not high and the color is dark at this time [1]. Therefore, the pH value of the solvent was set to 10.

### 3.2. Response Surface Test Optimization Results

#### 3.2.1. Response Surface Test Design and Results

Response surface test design and results are shown in Table S2.

#### 3.2.2. Regression Equation Fitting and Analysis of Variance (ANOVA)

A mathematical model with the regression equation was established by statistical analysis of the experimental data: extraction rate of QPI (%) = 73.48 + 1.61A + 2.55B − 0.16C + 3.66AB + 0.66AC +2.09BC − 9.49A^2^ − 1.05B^2^ − 4.61C^2^

ANOVA was performed for the regression model (Table S3). *p* < 0.01 indicated that the model equation was highly significant and the model’s lack of fit was insignificant. Therefore, the selected model was appropriate. The correlation coefficients were R^2^ = 0.9895 and R_Adj_^2^ = 0.9759, indicating that the equation fitted the test well, and can effectively reflect the relationship between each factor and its response value. Therefore, it was feasible to use this model equation to predict the process parameters of ultrasound-assisted quinoa protein extraction.

ANOVA of regression equation coefficients showed that the effect of ultrasonic time, the solid:liquid ratio on quinoa protein extraction rate, the interaction terms AB, and the interaction terms BC, were extremely significant; the effect of ultrasonic temperature on the quinoa protein extraction rate and the interaction terms AC was insignificant. The F value represents the degree of influence of ultrasonic time, solid-liquid ratio, and ultrasonic temperature on the extraction rate of QPI. A higher F value indicated greater influence. The order of influence of the three influencing factors on quinoa protein extraction rate, as inferred from the magnitude of F values, was solid-liquid ratio > ultrasonic time > ultrasonic temperature.

#### 3.2.3. Response Surface Analysis of Interaction of Various Factors

Response surface diagram and contour diagram of the interaction among ultrasonic time (A), solid-liquid ratio (B), and ultrasonic temperature (C) were prepared according to the regression equation (Figure 1E–G). A very steep curve was obtained for interactions between ultrasonic time and solid-liquid ratio, and solid-liquid ratio and ultrasonic temperature, indicating that these interactions had extremely significant effects on the extraction rate of QPI. A gentle curve obtained for the interaction between ultrasonic time and temperature indicated that these interactions did not have significant effects on the protein extraction rate. This was consistent with the results of ANOVA of regression equation.

The optimal process parameters of ultrasound-assisted quinoa protein extraction, determined by the mathematical model, were as follows: ultrasonic time 98.51 min, solid-liquid ratio 1:20, ultrasonic temperature 46.82 °C, and pH 10; the predicted extraction rate of QPI under these conditions was 75.87%. For actual operation, the following conditions were considered: ultrasonic time 99 min, solid-liquid ratio 1:20 g·mL^−1^, ultrasonic temperature 47 °C, and pH 10; under these conditions, the extraction rate could reach 74.67 ± 1.08% and the purity of QPI obtained was 87.17 ± 0.58%. The time taken by the ultrasound-assisted method for quinoa protein extraction was significantly lower than the time taken by the traditional alkaline extraction method. This is probably because during the ultrasound-assisted extraction process, the rapidly formed cavitation bubbles have mechanical, chemical, and thermal effects on the medium, which significantly increases the extraction rate of QPI [38].

### 3.3. Comparison of Ultrasound-Assisted Extraction and Traditional Alkaline Extraction of Quinoa Protein

**Figure 2:**
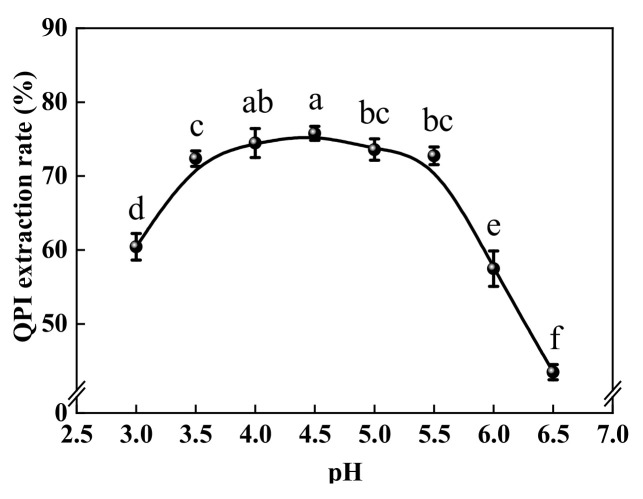
Isoelectric point of QPI. Different letters (a–f) represent significant differences (*p* < 0.05).

In an early phase of this study, single-factor and response surface test optimisation was carried out for the traditional alkaline extraction method using solid-liquid ratio, temperature, pH, and time as process parameters (Figure S1). The optimal conditions were as follows: time 139 min, solid-liquid ratio 1:29 *w:v*, temperature 56 °C, and pH 10; under these conditions, the protein extraction rate was 67.77 ± 1.50% and the purity of QPI was 82.63 ± 2.85% (Tables S4–S6). It was observed that the ultrasound-assisted extraction method significantly improved the extraction rate of QPI, i.e., by 10.18%, and increased the purity by 5.49% compared to the traditional alkaline extraction method. Moreover, using the ultrasound-assisted method, the extraction time and temperature were reduced by 40 min and 9 °C, respectively, thereby greatly improving the efficiency of quinoa protein extraction. Similar results were obtained by Li et al. [39] in the study of ultrasound-assisted extraction of capsicum seed protein. Thus, ultrasound-assisted quinoa protein extraction has a significant energy saving effect. In the pH range 3.0–6.5, the extraction rate of quinoa protein first increased, reached the maximum value at pH 4.5, and then decreased (Figure 2). Thus, the pI of QPI was 4.5, which is in agreement with the results obtained by Mir et al. [40] and Elsohaimy et al. [36].

### 3.4. Functional Properties of QPI

#### 3.4.1. Flexibility and Turbidity

**Figure 3:**
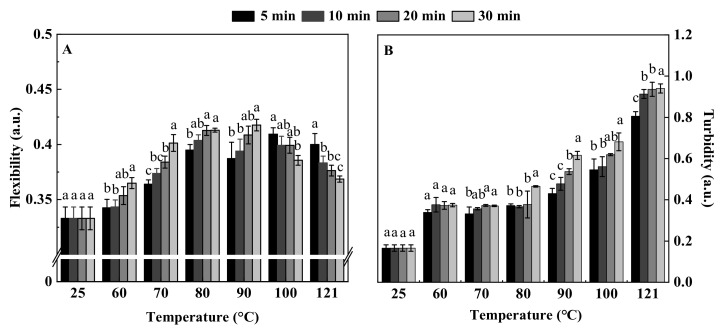
Effect of different heat treatment conditions on flexibility (**A**) and turbidity (**B**) of QPI. Different letters (a–c) indicate significant differences at same temperature (*p* < 0.05).

The flexibility of a protein can be understood as the ability of its structure to change when the external environment of the protein changes, reflecting the sensitivity of the protein structure to environmental changes [41]. It has received increasing attention because of its key role in determining the functional properties of proteins, especially the interfacial properties. Studies have shown that bovine serum albumin with higher flexibility is more likely to form a better viscoelastic protein film at the interface, thereby showing better emulsifying properties [42]. In this study, it was found that when the heat treatment time was certain, QPI flexibility increased and then decreased with the increase of temperature, reaching a maximum value of 0.42 at 90 °C, 30 min, which was significantly higher than that of untreated 27.27%. In the range of 60–90 °C, the flexibility of QPI gradually increased with the prolongation of heat treatment time. Previous studies have shown that rigid heat-resistant proteins reach the flexibility of unstable proteins at higher temperatures [43]. This is probably due to the fact that moderate heat treatment will destroy the covalent or non-covalent forces that maintain the rigid structure of the protein, such as van der Waals forces, hydrogen bonds, electrostatic interactions, disulfide bonds, hydrophobic interactions, etc., thereby increasing the flexibility of the protein [44]. When the temperature was 100 °C and 121 °C, QPI flexibility then gradually decreased with time and fell to the lowest value of 0.37 at 121 °C, 30 min, which was still higher than the untreated 12.12 % (Figure 3A). This is probably due to the excessive heating temperature that severely denatures the protein, forming a large number of insoluble agglomerates and decreasing solubility, which in turn leads to reduced flexibility [45].

The measurement of turbidity can directly reflect the dispersion state, aggregation state and particle size of protein particles in the solution [46]. In this study, it was found that the turbidity of QPI showed a gradual increase with the increase of time and temperature. At lower temperatures (60 °C and 70 °C), the effect of heat treatment time on QPI turbidity was not significant, while at higher temperatures the effect was significant, and QPI turbidity increased continuously with time, reaching a maximum value of 0.94 at 121 °C and 30 min, which was 4.53 times higher than that of untreated (Figure 3B). This is consistent with the result that heat treatment increases the turbidity of whey protein [47]. Previous studies have shown that the increase in turbidity is related to the formation of protein molecular aggregates [48]. In addition, higher aggregation rates and higher turbidity increases were also observed for longer microwave heating times for grass carp sarcoplasmic and myogenic fiber proteins [49]. Therefore, we speculate that the increase in turbidity may be due to the unfolding of QPI molecules, when treated at higher temperature and for a long time, the intramolecular and intermolecular interaction forces of proteins are enhanced, resulting in substantial aggregation of proteins [50].

#### 3.4.2. Nuclear Magnetic Resonance Hydrogen Spectroscopy (^1^H NMR)

**Figure 4:**
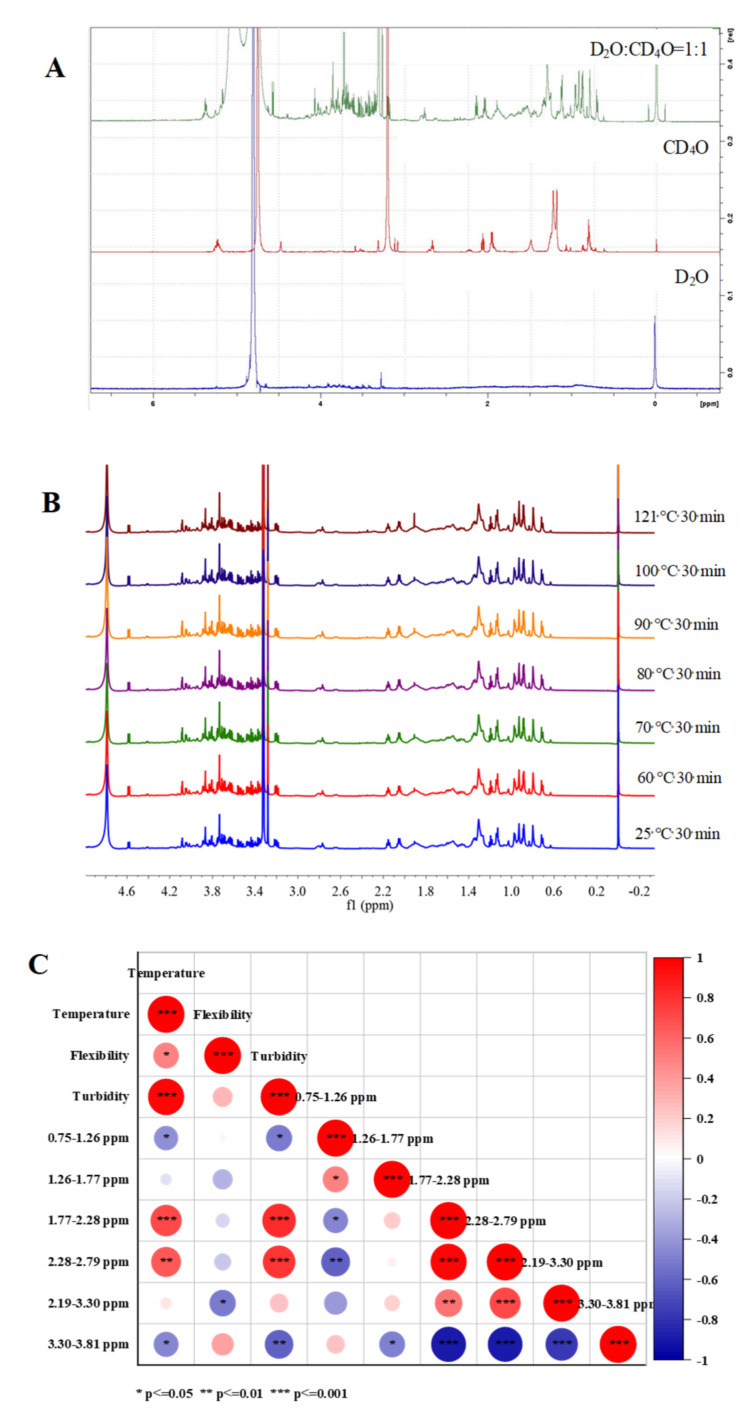
Effect of different solvents on dissolution of quinoa protein isolate (**A**), heat treatment temperatures on ^1^H NMR spectra of QPI (**B**), correlation between the influence of heat treatment on QPI ^1^H NMR spectrum and temperature, flexibility and turbidity (**C**).

A variety of solvents were first screened and three were finally identified for comparison, namely D_2_O, CD_4_O and a mixture of D_2_O:CD_4_O = 1:1. The best solution for QPI is D_2_O:CD_4_O = 1:1 solvent mixture (Figure 4A). Further study of the effect of different heat treatment temperatures on the ^1^H 600 MHz NMR spectra of QPI (Figure 4B). The peak at 0 ppm is the internal standard (sodium,2,2,3,3-tetradeuterio-3-trimethylsilylpropanoat -e), 4.50–4.80 ppm is the water peak, and 0.75–3.81 ppm is the amino characteristic range of protein [51,52]. The spectra of all QPIs in the range of 0.75 ppm to 3.81 ppm are very complete and clear, which is helpful for further data processing and analysis. In this study, the 1H 600 MHz NMR spectra of QPI were subjected to a series of operations such as phase correction, baseline correction, calibration, peak calibration, integration and normalization in the range of 0.75–3.81 ppm in units of 0.51 ppm to obtain the NMR integral spectra (Figure S2). The results showed that only the normalized relative percentages in the range of 3.30–3.81 ppm were dependent on the heat treatment temperature of QPI, which gradually decreased with increasing temperature in the range of 60–121 °C, and had a minimum value of 34.97 ± 0.82% at the heat treatment condition of 121 °C for 30 min, which was significantly lower than the untreated 8.46% (*p* < 0.05). Its variation in the interval from 0.75 to 1.26 ppm was not only dependent on temperature, but also at 121 °C, 30 min had a minimum value of 23.25 ± 0.34%, significantly lower than the untreated 2.47% (*p* < 0.05). Its variation in the interval of 1.77–2.28 ppm, 2.28–2.79 ppm and 2.79–3.30 ppm was not dependent on temperature and reached maximum values of 10.85 ± 0.07%, 2.26 ± 0.38% and 5.42 ± 0.96% at the heat treatment condition of 121 °C for 30 min, respectively, which were significantly higher than the untreated 24.57%, 4.51 times and 7.54 % (*p* < 0.05) (Table 1). This indicates that 0.75–1.26 ppm, 3.30–3.81 ppm, 1.77–2.28 ppm, 2.28–2.79 ppm and 2.79–3.30 ppm are all susceptible to heat treatment.

Heat treatment temperature has a significant effect on the 1H NMR spectrum, turbidity and flexibility of QPI (Figure 4C). The ^1^H NMR of 1.77–2.28 ppm and 2.28–2.79 ppm showed highly significant positive correlations (*p* < 0.01) with heat treatment temperature and turbidity, and 3.30–3.81 ppm showed highly significant negative correlations (*p* < 0.01) with turbidity and significant negative correlations (*p* < 0.05) with heat treatment temperature. In addition, 0.75–1.26 ppm also had a significant negative correlation with temperature (*p* < 0.05), while flexibility had a significant positive correlation with temperature (*p* < 0.05) and turbidity had a highly significant positive correlation with temperature (*p* < 0.001). This suggests that heat treatment affects the protein characteristic 1.77 to 2.79 ppm region of quinoa, which in turn alters the turbidity of the QPI. However, studies on the influence of heat treatment on 1H NMR spectra and microscopic substances are still in the initial stage, and the specific mechanisms and principles are not well understood, and further in-depth studies are needed.

### 3.5. Hydrolysis Degree during In Vitro Digestion

**Figure 5:**
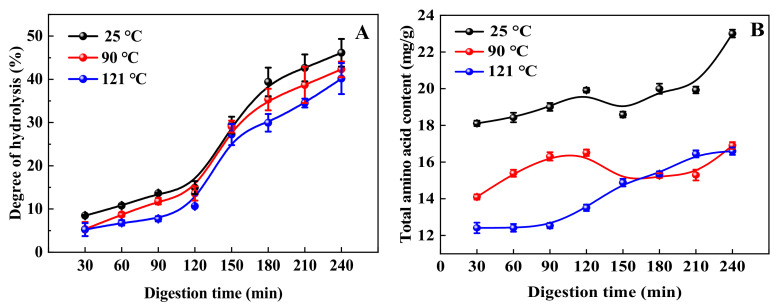
Effect of different heat treatment temperatures on the degree of hydrolysis (**A**) and total amino acid content (**B**) of QPI during in vitro digestion. Different letters indicate significant differences (*p* < 0.05).

Degree of hydrolysis (DH) is defined as the proportion of cleaved peptide bonds in the protein hydrolysate [53]. In this study, the trend of hydrolysis of QPI after heat treatment was similar to that of untreated QPI, and the hydrolysis of QPI after heat treatment at 90 and 121 °C for 30 min was lower than that of untreated QPI during the whole digestion process, among which the hydrolysis of QPI after treatment at 121 °C was the smallest, which was 25.53% after simulated in vitro digestion for 240 min, significantly lower than that of untreated QPI at 20.64% (Figure 5A). Previous studies found that the hydrolysis of soybean isolate protein (SPI) decreased under heat treatment conditions greater than 85 °C [54]. Reduction in protein digestibility of QPI after heating treatment has generally been attributed to the formation of cross-linked protein polymers, which are resistant to proteolysis [55]. Therefore, after heat treatment of the QPI suspension, pepsin became less effective in enzymatic digestion and its digestibility was lower than that of untreated protein [6]. This is consistent with the results of reduced digestibility of chicken breast proteins after heat treatment at 121 °C [56]. Previous studies have shown that heat treatment is an important process for the complete digestion of food proteins. These processes significantly affect the protein structure and thus its digestive resistance [57]. Therefore, we speculate that the QPIs treated at 90 °C and 121 °C may have structures that are difficult to degrade enzymatically and may inhibit the activity of digestive enzymes.

### 3.6. Total Amino Acid Content during In Vitro Digestion

This study found that the content of each amino acid at the end point of pepsin digestion (120 min) was significantly lower than that of trypsin digestion end point (240 min) at the same temperature (Figure 5B). The amino acid content increased gradually with the prolongation of digestion time, and the amino acid content in the trypsin digestion stage was significantly higher than that in the pepsin digestion stage. The content of amino acids in the in vitro digestion products of untreated QPI was higher than that of the heat treated products. The content of amino acids in the in vitro digestion products of QPI after treatment at 121 °C was the lowest, and the content at the end of digestion was 16.60 mg/g (Table 2), which was significantly lower than that of untreated 27.85%. Previous studies have found that the amino acid content of heat-treated king oyster mushroom protein was significantly reduced after digestion in vitro [58]. Amino acid content of in vitro digested gluten protein was significantly reduced after microwave heating [59]. Furthermore, Giménez et al. [60] indicated that low levels of His, Pro and Ala in protein hydrolyzates were associated with a decrease in their antioxidant potential. Similar results were found in soy protein isolates [61]. In this study, the contents of His, Pro and Ala in the in vitro digestion products of QPI treated at 90 °C and 121 °C were significantly lower than those of untreated. Therefore, we speculate that the high heating temperature might cause serious damage to amino acids and irreversible decomposition, resulting in the reduction of content. This result confirms the reason why heat treatment leads to a decrease in the antioxidant activity of QPI in vitro.

## 4. Conclusions

In this study, the effects of ultrasonic-assisted and traditional alkali-dissolving and acid-precipitation methods on the extraction of QPI were compared. The effect of heat treatment on the functional properties and in vitro digestion properties of QPI were studied. The results indicate that the factors influencing the extraction rate of QPI by ultrasound-assisted alkaline extraction method were solid-liquid ratio > ultrasonic time > ultrasonic temperature, in order of their influence. The optimal conditions for extraction were as follows: ultrasonic time 99 min, solid-liquid ratio 1:20 *w:v*, ultrasonic temperature 47 °C, and pH 10. Under these conditions, the extraction rate reached 74.67 ± 1.08% and the purity of QPI obtained was 87.17 ± 0.58%. In comparison with the traditional alkaline dissolution and acid precipitation method, the extraction rate and purity of QPI extracted by ultrasound-assisted method were increased by 10.18% and 5.49%, respectively. The pI of QPI is 4.5. Heat treatment had a significant effect on the ^1^H NMR spectrum, turbidity and flexibility of QPI. Heat treatment changed the turbidity of QPI by affecting the 1.77 to 2.79 ppm region in the ^1^H NMR spectrum of QPI. After heat treatment, the degree of hydrolysis and amino acid content of QPI in vitro digestion decreased. The results of this study provide a basis for processing and utilization of quinoa protein isolate.

## Figures and Tables

**Table 1:** Effect of different heat treatment temperatures on the relative percentage content of QPI’s ^1^H NMR integrated spectra after normalization.

Temperature (°C)	Relative Percentage Content (%)					
	0.75–1.26 ppm	1.26–1.77 ppm	1.77–2.28 ppm	2.28–2.79 ppm	2.79–3.30 ppm	3.30–3.81 ppm
25	23.84 ± 0.19 ^ab^	23.80 ± 0.16 ^a^	8.71 ± 0.19 ^c^	0.41 ± 0.07 ^bc^	5.04 ± 0.29 ^ab^	38.20 ± 0.07 ^d^
60	23.30 ± 0.09 ^c^	22.38 ± 0.06 ^d^	8.64 ± 0.04 ^c^	0.47 ± 0.13 ^bc^	4.29 ± 0.29 ^b^	40.93 ± 0.30 ^a^
70	23.58 ± 0.14 ^bc^	22.778 ± 0.04 ^c^	8.758 ± 0.07 ^c^	0.38 ± 0.16 ^bc^	4.23 ± 0.25 ^b^	40.30 ± 0.29 ^ab^
80	23.96 ± 0.05 ^a^	23.34 ± 0.12 ^b^	8.84 ± 0.07 ^c^	0.27 ± 0.08 ^c^	4.12 ± 0.35 ^b^	39.46 ± 0.25 ^c^
90	23.47 ± 0.06 ^bc^	22.91 ± 0.01 ^c^	8.73 ± 0.04 ^c^	0.52 ± 0.06 ^bc^	4.38 ± 0.06 ^b^	39.99 ± 0.08 ^bc^
100	23.47 ± 0.17 ^bc^	23.47 ± 0.05 ^b^	9.27 ± 0.13 ^b^	0.75 ± 0.13 ^b^	4.40 ± 0.10 ^b^	38.64 ± 0.11 ^d^
121	23.25 ± 0.34 ^c^	23.24 ± 0.23 ^b^	10.85 ± 0.07 ^a^	2.26 ± 0.38 ^a^	5.42 ± 0.96 ^a^	34.97 ± 0.82 ^e^

Different letters (^a–e^) represent significant differences in the same column (*p* < 0.05).

**Table 2:** Total amino acid content in vitro digestion products of quinoa protein isolate after different heat treatment.

Type		Content (mg·g^−1^)					
		25 °C		90 °C		121 °C	
		120 min	240 min	120 min	240 min	120 min	240 min
Essential Amino Acids	Thr	0.82 ± 0.02	0.94 ± 0.04	0.68 ± 0.07	0.70 ± 0.02	0.54 ± 0.07	0.68 ± 0.03
	Met	0.48 ± 0.03	0.55 ± 0.01	0.39 ± 0.04	0.39 ± 0.04	0.31 ± 0.04	0.39 ± 0.03
	Val	1.12 ± 0.09	1.29 ± 0.18	0.92 ± 0.10	0.94 ± 0.05	0.74 ± 0.14	0.93 ± 0.05
	Lys	1.24 ± 0.12	1.44 ± 0.08	1.03 ± 0.09	1.05 ± 0.10	0.82 ± 0.04	1.03 ± 0.12
	Leu	1.76 ± 0.11	2.04 ± 0.14	1.45 ± 0.20	1.48 ± 0.08	1.16 ± 0.12	1.45 ± 0.08
	Ile	1.03 ± 0.04	1.19 ± 0.15	0.85 ± 0.07	0.87 ± 0.08	0.68 ± 0.06	0.85 ± 0.06
	Phe	1.02 ± 0.09	1.18 ± 0.12	0.84 ± 0.04	0.86 ± 0.08	0.67 ± 0.07	0.84 ± 0.06
Non- essential Amino Acids	Asp	1.90 ± 0.01	2.19 ± 0.07	1.58 ± 0.09	1.62 ± 0.06	1.27 ± 0.07	1.58 ± 0.02
	Tyr	0.80 ± 0.08	0.93 ± 0.05	0.66 ± 0.02	0.67 ± 0.09	0.53 ± 0.10	0.67 ± 0.06
	Ser	0.99 ± 0.01	1.15 ± 0.12	0.83 ± 0.11	0.85 ± 0.06	0.66 ± 0.09	0.83 ± 0.03
	Glu	3.10 ± 0.24	3.52 ± 0.19	2.62 ± 0.14	2.68 ± 0.12	2.11 ± 0.25	2.61 ± 0.19
	Gly	1.03 ± 0.12	1.20 ± 0.05	0.86 ± 0.04	0.88 ± 0.08	0.68 ± 0.07	0.86 ± 0.04
	Ala	1.02 ± 0.09	1.18 ± 0.16	0.84 ± 0.07	0.86 ± 0.07	0.67 ± 0.08	0.84 ± 0.04
	Cys	0.23 ± 0.01	0.28 ± 0.02	0.16 ± 0.02	0.18 ± 0.08	0.16 ± 0.05	0.20 ± 0.04
	Pro	0.64 ± 0.08	0.70 ± 0.03	0.52 ± 0.01	0.56 ± 0.08	0.45 ± 0.05	0.55 ± 0.04
	His	0.66 ± 0.05	0.77 ± 0.08	0.54 ± 0.04	0.55 ± 0.07	0.43 ± 0.05	0.55 ± 0.04
	Arg	2.10 ± 0.08	2.44 ± 0.07	1.74 ± 0.19	1.77 ± 0.09	1.39 ± 0.10	1.74 ± 0.11
	TAA	19.92 ± 0.08	23.00 ± 0.09	16.51 ± 0.08	16.90 ± 0.07	13.27 ± 0.09	16.60 ± 0.06

## Data Availability

No new data were created or analyzed in this study. Data sharing is not applicable to this article.

## Supplementary Materials

The following supporting information can be downloaded at: https://www.mdpi.com/article/10.3390/foods11050771/s1, Figure S1: The effects of time (A), solid-liquid ratio (B), temperature (C), pH (D) and the response surface methodology and contour plots for the effects of various factors (E, F, G) on the extraction rate of QPI. Different letters represent significant differences (*p* < 0.05). Figure S2: Effect of different heat treatment temperatures on 1H NMR integrated spectra of QPI.;Table S1: Codes and levels of factors for response surface methodology experiments. (Ultrasonic); Table S2: Central composite arrangement for independent variables A (Ultrasonic time, min), B (Solid-liquid ratio, g·mL^−1^), C (Ultrasonic temperature, °C) and their response (QPI extraction rate, %); Table S3: Regression equation analysis of variance. (Ultrasonic); Table S4: Codes and levels of factors for response surface methodology experiments. (Alkali-solution and acid-isolation); Table S5: Central composite arrangement for independent variables A (Time, min), B (Solid-liquid ratio, g·mL^−1^), C (Temperature, °C) and their response (QPI extraction rate, %); Table S6: Regression equation analysis of variance. (Alkali-solution and acid-isolation).
