# Supplementary material for: Ultrasonic Assisted Extraction of Quinoa (Chenopodium quinoa Willd.) Protein and Effect of Heat Treatment on Its In Vitro Digestion Characteristics
Source: Foods. 2022 Mar 7;11(5):771. doi: 10.3390/foods11050771 (PMC8909454; doi:10.3390/foods11050771)
Supplement: Supplementary file 1 [file foods-11-00771-s001.zip › foods-1609553-supplementary.pdf]

Supplementary Materials

Ultrasonic assisted extraction of quinoa (*Chenopodium quinoa* Willd.) protein and effect of heat treatment on its in vitro digestion characteristics

*Xingfen He, Bin Wang, Baotang Zhao, Fumin Yang**

College of Food Science and Engineering, Gansu Agricultural University, Lanzhou 730070, The People's Republic of China

*Corresponding author
Fumin Yang
College of Food Science and Engineering, Gansu Agricultural University, Yingmen Village No.1, Lanzhou 730070, The People's Republic of China
Tele-phone: +86 13893337478;
E-mail address: yfumin@163.com

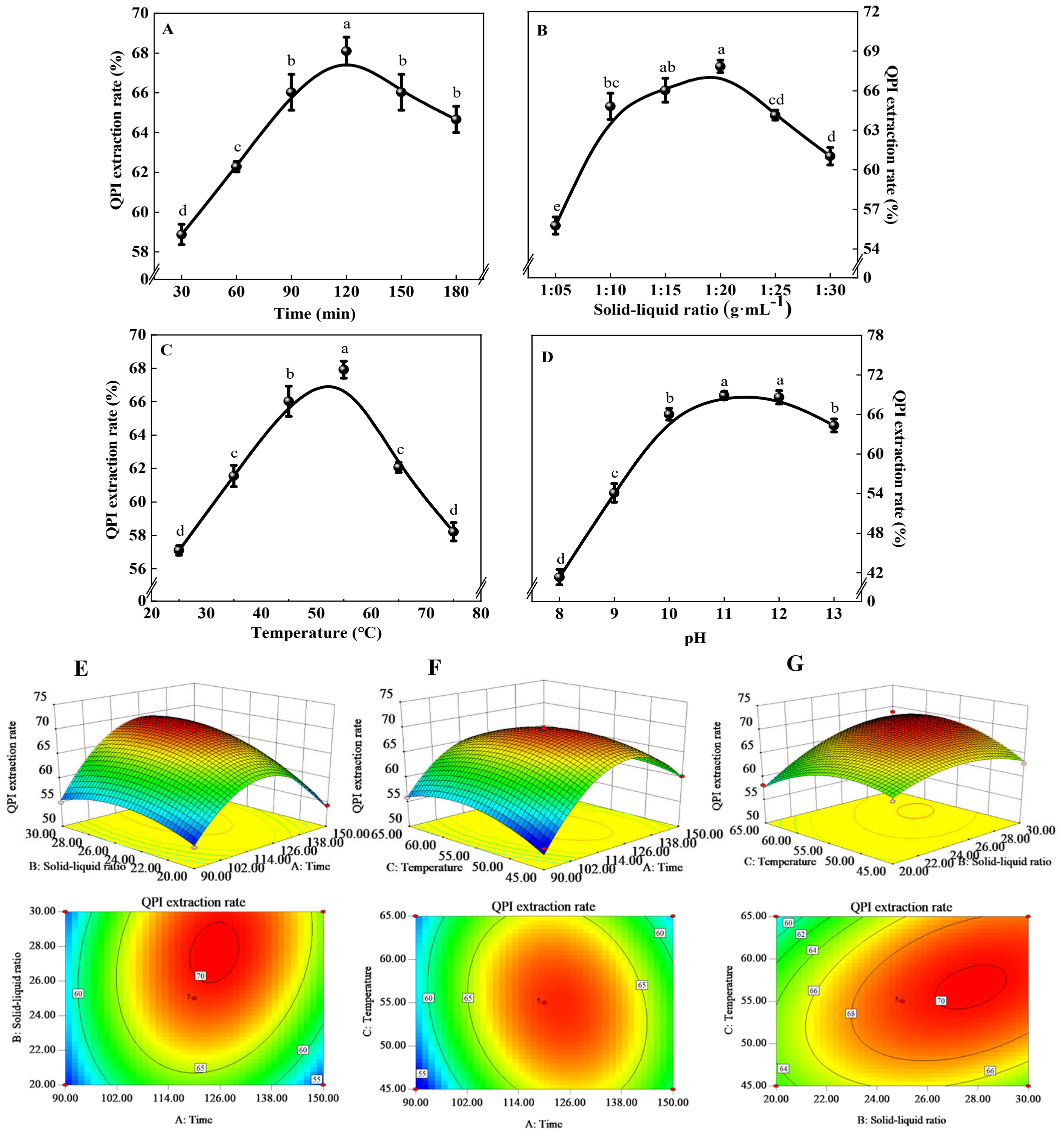

Figure S1. The effects of time (A), solid-liquid ratio (B), temperature (C), pH (D) and the response surface methodology and contour plots for the effects of various factors (E, F, G) on the extraction rate of QPI. Different letters represent significant differences (P<0.05).

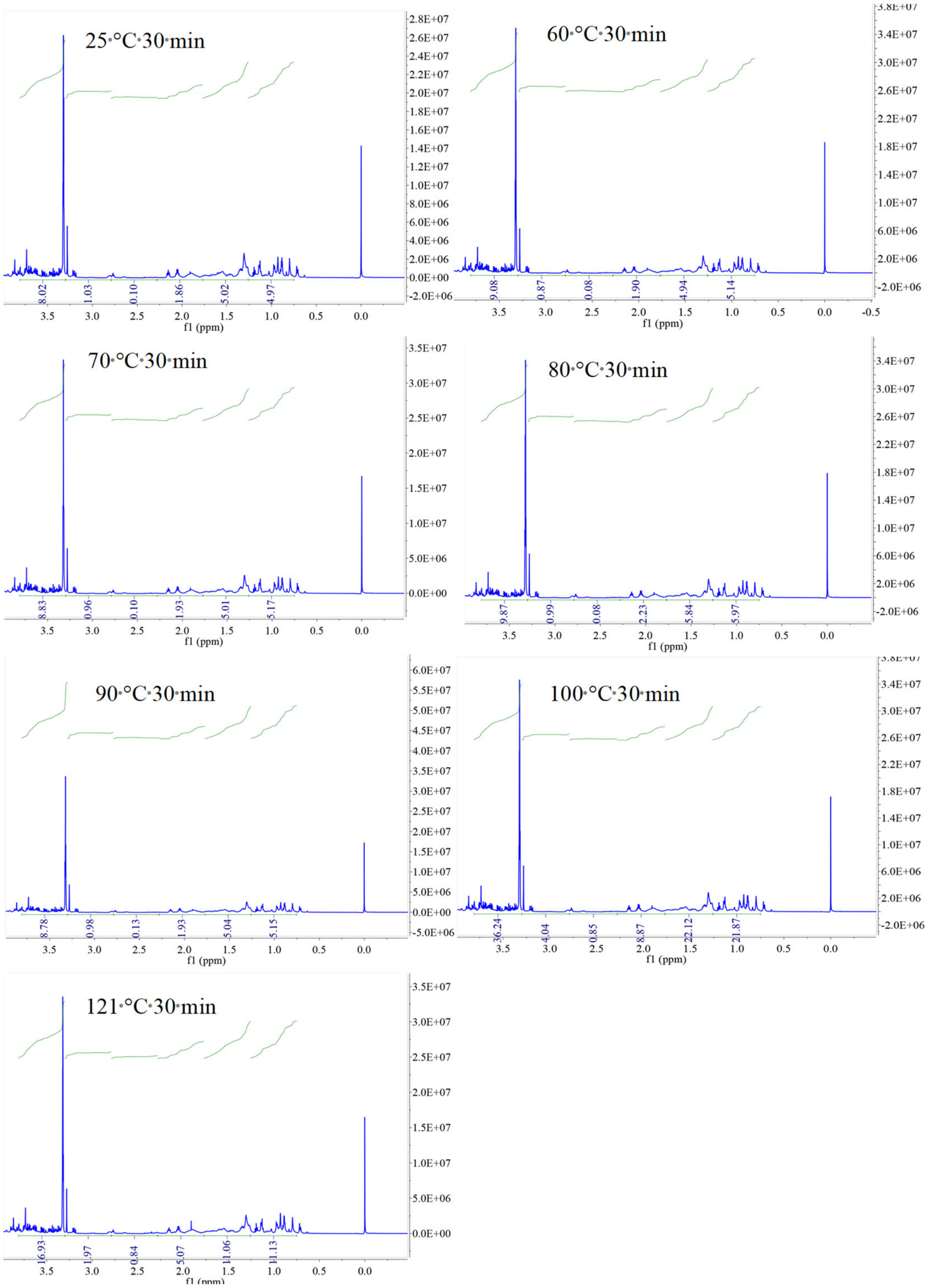

Figure S2. Effect of different heat treatment temperatures on ^{1}H NMR integrated spectra of QPI.

Table S1. Codes and levels of factors for response surface methodology experiments. (Ultrasonic)

Level	Factor		
	A Ultrasonic time (min)	*B* Solid-liquid ratio (v/w)	*C* Ultrasonic temperature (℃)
-1	60	1:10	35
0	90	1:15	45
1	120	1:20	55

Table S2. Central composite arrangement for independent variables A (Ultrasonic time, min), B (Solid-liquid ratio, g·mL^{-1}), C (Ultrasonic temperature, ℃) and their response (QPI extraction rate, %).

Run	Response			
	A	B	C	Experimental
	Ultrasonic time (min)	Solid-liquid ratio (v/w)	Ultrasonic temperature (℃)	QPI extraction rate(%)
1	-1	-1	0	62.10
2	1	-1	0	58.51
3	-1	1	0	60.07
4	1	1	0	71.10
5	-1	0	-1	60.03
6	1	0	-1	61.43
7	0	0	1	56.03
8	0	0	1	60.07
9	0	-1	-1	67.33
10	0	1	-1	68.07
11	0	-1	1	63.40
12	0	1	1	72.51
13	0	0	0	73.33
14	0	0	0	72.33
15	0	0	0	73.84
16	0	0	0	73.73
17	0	0	0	74.18

Table S3. Regression equation analysis of variance. (Ultrasonic)

Source	Sum of Square	df	Mean Square	F Value	p-yalue Prob>F	Significant
A-Ultrasonic time(min)	20.77	1	20.77	20.96	0.0026	**
B-Solid-liquid ratio(v/w)	52.06	1	52.06	52.54	0.0002	**
C-Ultrasonic tempera-ture(°C)	2.94	1	2.94	2.97	0.1285	
AB	53.51	1	53.51	54.00	0.0002	**
AC	1.73	1	1.73	1.74	0.2281	
BC	17.52	1	17.52	17.68	0.0040	**
A^2	378.82	1	378.82	382.30	<0.0001	**
B^2	4.64	1	4.64	4.68	0.0672	
C^2	89.31	1	89.31	90.13	<0.0001	**
Model	651.59	9	72.40	73.06	<0.0001	**
Residual	6.94	7	0.99			
Lack of Fit	4.90	3	1.63	3.20	0.1452	
Pure Error	2.04	4	0.51			
Cor Total	658.52	16				

Note: **. Correlation is significant at the 0.01 level; *. Correlation is significant at the 0.05 level.

A mathematical model with the regression equation was established by statistical analysis of the experimental results: extraction rate of QPI (%) $=73.48+1.61A+2.55B-0.61C+3.66AB+0.66AC+2.09BC-9.49A^2-1.05B^2-4.61C^2$

Table S4. Codes and levels of factors for response surface methodology experiments. (Alkali-solution and acid-isolation)

Level	Factor		
	A Time (min)	*B* Solid-liquid ratio (v/w)	*C* Temperature (°C)
-1	90	1:20	45
0	120	1:25	55
1	150	1:30	65

Table S5. Central composite arrangement for independent variables A (Time, min), B (Solid-liquid ratio, g·mL^{-1}), C (Temperature, ℃) and their response (QPI extraction rate, %)

Run	Response			
	A	B	C	Experimental
	Time (min)	Solid-liquid ratio (v/w)	Temperature (℃)	QPI extraction rate(%)
1	90	20	55	54.10
2	150	20	55	54.51
3	90	30	55	55.07
4	150	30	55	64.07
5	90	25	45	54.03
6	150	25	45	60.44
7	120	25	65	56.03
8	150	25	65	55.07
9	120	20	45	62.33
10	120	30	45	63.03
11	120	20	65	58.40
12	120	30	65	68.92
13	120	25	55	69.21
14	120	25	55	68.25
15	120	25	55	69.88
16	120	25	55	69.92
17	120	25	55	70.10

Table S6. Regression equation analysis of variance. (Alkali-solution and acid-isolation).

Source	Sum of Square	df	Mean Square	F Value	p-yalue Prob>F	Significant
A-Ultrasonic time(min)	27.57	1	27.57	24.43	0.0017	**
B-Solid-liquid ratio(v/w)	59.08	1	59.08	52.34	0.0002	**
C-Ultrasonic temperature(℃)	0.25	1	0.25	0.22	0.6538	
AB	18.46	1	18.46	16.35	0.0049	**
AC	13.58	1	13.58	12.03	0.0104	*
BC	24.08	1	24.08	21.33	0.0024	**
A^2	392.70	1	392.70	347.89	<0.0001	**
B^2	34.91	1	34.91	30.93	0.0008	**
C^2	49.42	1	49.42	43.78	0.0003	**
Model	656.16	9	72.91	64.59	<0.0001	**
Residual	7.90	7	1.13			
Lack of Fit	5.58	3	1.86	3.21	0.1449	
Pure Error	2.32	4	0.58			
Cor Total	664.06	16				

Note: **. Correlation is significant at the 0.01 level; *. Correlation is significant at the 0.05 level.

A mathematical model with the regression equation was established by statistical analysis of the experimental results: extraction rate of QPI (%) $=69.47+1.86A+2.72B-0.18C+2.15AB-1.84AC+2.45BC-9.66A^2-2.88B^2-3.43C^2$
$R^2=0.9881$ $R_{adj}^2=0.9728$.
